# Association between four insulin resistance surrogates and the risk of esophageal cancer: a prospective cohort study using the UK Biobank

**DOI:** 10.1007/s00432-024-05919-8

**Published:** 2024-08-24

**Authors:** Chuang Yang, Wenke Cheng, Patrick S. Plum, Jeanette Köppe, Ines Gockel, René Thieme

**Affiliations:** 1 Department of Visceral, Transplant, Thoracic and Vascular Surgery, University Hospital Leipzig, Liebigstr. 20, D-04103 Leipzig, Germany; 2 Medical Faculty, University of Leipzig, Leipzig, Germany; 3 Institute of Biostatistics and Clinical Research, University of Muenster, Muenster, Germany

**Keywords:** Esophageal cancer, Insulin resistance, TyG index, TyG-BMI, TG/HDL-C, METS-IR

## Abstract

**Purpose:**

This study explored the association between triglyceride-glucose (TyG), TyG index with body mass index (TyG-BMI), triglyceride/high-density lipoprotein cholesterol ratio (TG/HDL-C), metabolic score for insulin resistance (IR) (METS-IR) and the risk of esophageal cancer.

**Methods:**

A total of 388,900 participants from the United Kingdom Biobank from 2006 to 2010 were included. Fine-Gray models, restricted cubic spline (RCS), and receiver operating characteristic (ROC) curves were used to assess the association between the four IR surrogates and the risk of esophageal cancer, specifically, esophageal adenocarcinoma (EAC) and esophageal squamous cell carcinoma (ESCC).

**Results:**

Ten years after recruitment, 0.16% (95%CI 0.11–0.26%) had esophageal cancer and 4.17% (95%CI 3.86–4.46%) are deceased. For each standard deviation increase in the TyG index, TyG-BMI, TG/HDL-C, and METS-IR, the risk of EAC increased by Hazard ratios (HR)1.16, 1.37, 1.08, and 1.36, respectively (all *P* < 0.05), while the risk of ESCC decreased by HRs 0.80, 0.67, 0.77, and 0.65, respectively. RCS analysis indicated that most relationships were nonlinear (*P* < 0.05). ROC curves showed that METS-IR had a more robust diagnostic efficacy than TyG, TyG-BMI, and TG/HDL-C.

**Conclusion:**

TyG index, TyG-BMI, TG/HDL-C, and METS-IR were closely associated with the risk of EAC and ESCC. Additionally, METS-IR surpassed the other three IR indices in predicting and diagnosing the risks of EAC and ESCC. The METS-IR is expected to become a more effective metric for identifying populations at early risk of esophageal cancer and for improving risk stratification.

**Supplementary Information:**

The online version contains supplementary material available at 10.1007/s00432-024-05919-8.

## Introduction

Despite ongoing advances in diagnosis and treatment, esophageal cancer remains a serious global threat to human health. According to global cancer statistics, there were 604,000 new cases of esophageal cancer in 2020, ranking it seventh among all cancers in incidence, and 544,000 deaths, ranking esophageal cancer sixth among all cancers in terms of mortality (Sung et al. 2021). This indicates that in 2020, one in every eighteen cancer-related deaths was caused by esophageal cancer, with 70% of the cases occurring in men (Sung et al. 2021). Esophageal squamous cell carcinoma (ESCC) and esophageal adenocarcinoma (EAC) are the two predominant esophageal cancer histological entities. Although EAC accounts for only 14% of the total global cases of esophageal cancer, it represents two-thirds of the cases in high-income countries, and its incidence is continuously increasing (Morgan et al. 2022).

Metabolic syndrome (MetS) comprises a group of metabolic disorders characterized by obesity, hypertension, high blood sugar levels, and abnormal lipid levels. Previous studies have revealed a close association between esophageal cancer and MetS (Zhang et al. 2021; Lee et al. 2022b). Among them, it was recently suggested that MetS is associated with an increased risk of EAC (odds ratio [OR]: 1.19, 95% confidence interval [CI] 1.10–1.28), with no correlation observed with the risk of ESCC (OR: 1.09; 95%CI 0.89–1.34) (Zhang et al. 2021).

Insulin resistance (IR), a crucial feature of MetS and obesity, is increasingly recognized as a key factor in cancer development. Substantial evidence indicates that IR is associated with the occurrence of various cancers, including lung, prostate, colorectal, and breast cancers (Saboori et al. 2019; Farahani et al. 2020; Lee et al. 2022a; Liu et al. 2023b, 2024), suggesting that IR may serve as an effective tool for identifying individuals at risk for cancer.

The hyperinsulinemic-euglycemic clamp test is considered the “gold standard” for evaluating IR (Liu et al. 2023b). However, because of the equipment complexity, operational intricacies, and invasiveness, the hyperinsulinemic-euglycemic clamp test is not commonly used in clinical practice (Liu et al. 2023b). Consequently, more practical surrogate markers for IR have been developed to replace direct insulin measurements. These included the triglyceride-glucose (TyG) index (Ramdas Nayak et al. 2022), TyG index combined with body mass index (BMI) (TyG-BMI) (Er et al. 2016), triglyceride-to-high-density lipoprotein cholesterol ratio (TG/HDL-C) (Young et al. 2019), and the metabolic score for IR (METS-IR) (Bello-Chavolla et al. 2018). These non-insulin-based fasting IR indices are calculated based on lipid parameters and Body Mass Index (BMI), offering a convenient and rapid means of assessing IR.

Previous studies have explored the association between IR surrogate markers and cancer risk, consistently indicating that a higher risk of cancer is associated with elevated IR surrogates (Liu et al. 2022; Wang et al. 2022a, b, 2024).

However, whether different IR surrogates are associated with the risk of esophageal cancer remains unclear. Therefore, in this study, we aimed to systematically examine the association between the four IR surrogates and the overall risk of esophageal cancer using United Kingdom Biobank (UKB) data, including TyG, TyG-BMI, TG/HDL-C, and METS-IR. Additionally, we explored the associations between these four IR surrogates and different esophageal cancer subtypes (EAC and ESCC).

## Materials and methods

### Study design and population

The UKB is a continuing prospective community-based cohort study that enrolled nearly 500,000 participants aged 37–73 years between 2006 and 2010. It collected sociodemographic information, biological samples, disease history, and questionnaires during recruitment from 22 assessment centers across England, Wales, and Scotland. Ethical approval for the study was obtained from the North West Multi-Center Research Ethics Committee, and all participants provided written informed consent. More comprehensive information regarding the UKB has been previously provided (Sudlow et al. 2015).

### Data collection and definitions

Demographic information on age, sex, ethnicity, Townsend deprivation index, physical activity (assessed by the Metabolic Equivalent of Task [MET]), smoking and alcohol status, BMI, history of diabetes mellitus (DM), hypertension and cardiovascular disease (CVD) history, lipid-lowering drug use, insulin use, and diet were collected using a baseline touchscreen questionnaire. The diet questionnaire measured the consumption of nine food components: processed meat, red meat, fish, milk, spreads, cereals, added salt, water, and fruits and vegetables, with scores ranging from 0 to 9 points (Table S1) (Petermann-Rocha et al. 2021).

Blood samples were collected from all participants at the time of enrollment and analyzed within 24 h. Blood specimens were collected randomly and fasting time was recorded during collection. Data on several blood biochemistry indicators, including triglyceride, fasting plasma glucose (FPG), total cholesterol, and fasting time before sampling, were collected. The following formula was used to calculate IR surrogate indices: TyG index = ln [triglyceride (mg/dL) × FPG (mg/dL) / 2] (Cheng et al. 2022). Additionally, TyG-BMI = TyG × BMI (Bala et al. 2019), TG/HDL-C = triglycerides divided by high-density lipoprotein cholesterol (Liu et al. 2022), and METS-IR = ln [(2 × FPG) + triglycerides] × BMI/ln (HDL-C) (Wang et al. 2024).

### Outcome assessment

The study outcome was the incident diagnosis of any type of esophageal cancer in the UKB, recorded using the International Statistical Classification of Diseases and Related Health Problems, Tenth Revision (ICD-10) codes (Table S2) (Listed 2009). The histology was classified according to the International Classification of Diseases for Oncology (3rd Edition) in the UK Biobank (https://biobank.ndph.ox.ac.uk/showcase/ukb/docs/ICDcancermorph.pdf).

Participants were followed-up from the date of recruitment until the date of esophageal cancer diagnosis, date of death, or last recorded date of any cancer event (June 1, 2022), whichever came first.

#### Selection criteria

**Fig. 1:**
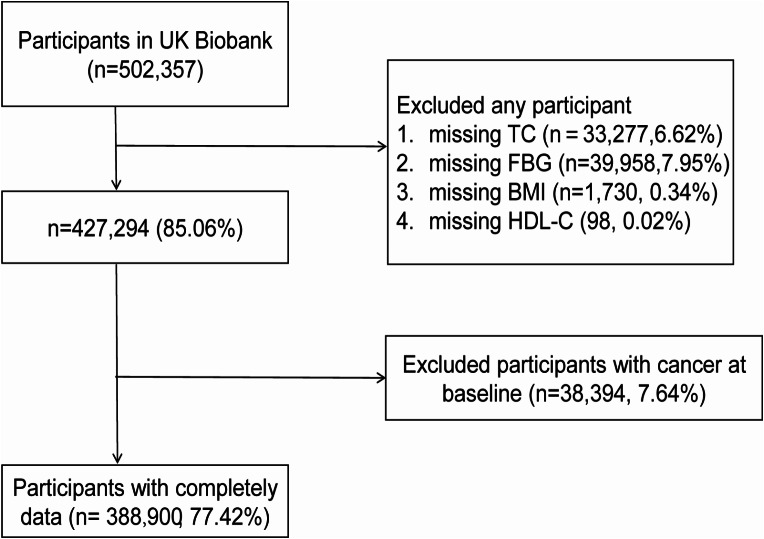
Flow diagram of participant selection

In this study, participants lacking triglyceride data at recruitment (*n* = 33,277), FBG (Fasting Blood Glucose) data (*n* = 39,958), BMI data (*n* = 1,730), HDL-C data (*n* = 98), and those diagnosed with any type of cancer before enrollment (*n* = 38,394) were excluded. Finally, 388,900 participants with complete exposure data were retrospectively included in this study (Fig. 1).

### Missing values

For the covariates, missing values of continuous variables were imputed using mean imputation, whereas missing values of categorical variables were imputed using mode imputation.

### Statistical analysis

#### Descriptive analysis

Median and interquartile range were used to describe continuous variables, frequencies (N), and percentages (%). The data from the four IR surrogates were normalized to Z-scores and then used for subsequent analysis.

#### Primary endpoint – univariate analyses

The primary endpoint was defined as the time from recruitment to first coded esophageal cancer, where death was considered as a competing risk event. Therefore, the cumulative incidence rates for esophageal cancer (including total esophageal cancer, EAC, and ESCC) were given by Aalen-Johansen estimates with 95% CI and presented separately for the quartiles of the TyG index, TyG-BMI, TG/HDL-C, and METS-IR. And the P values were determined by Gray’s test.

### Primary endpoints – multivariable analyses

To investigate the relationship between the four IR surrogates and the risk of esophageal cancer, Fine and Gray regression models were used to assess the sub-distributional hazard ratios (HRs) and 95% CIs or the higher quartiles compared with the lowest quartile, as well as for each standard deviation (SD) increment of the TyG index, TyG-BMI, TG/HDL-C, and METS-IR for all endpoints. Linear trends across the four IR surrogate quartiles were evaluated using the median value within each quartile as a continuous variable.

Two models determining sub-distributional HRs were constructed to account for potential confounders of esophageal cancer. Model 1 was adjusted for age and sex, and Model 2 was further adjusted for ethnicity, Townsend deprivation index, MET, smoking and alcohol status, history of DM, hypertension, insulin, fasting time, and diet score. The selection of covariates was based on established a priori knowledge, and a directed acyclic graph (Fig. S1) was employed to determine their inclusion (Tennant et al. 2021).

### Sensitivity analysis for primary endpoints

As a sensitivity analysis, we excluded participants who developed esophageal cancer within the first 2 years of follow-up to mitigate the potential for reverse causality. Next, we excluded participants with missing values for any covariates to examine the impact of imputation on the results. Subsequently, we utilized multiple imputations to fill in the missing values, resulting in five complete datasets and separately calculated the association between exposure and outcome for each dataset. We compared the final results with those obtained using the mean and mode imputations to assess the influence of different imputation methods on the effects of the four IR surrogates on the outcomes.

### Explorative analyses

For further explorative analysis, cause-specific hazard ratios for esophageal cancer were modeled using Cox proportional hazard models. Subsequently, multivariate restricted cubic spline (RCS) analysis with three knots (10th, 50th, and 90th percentiles) was used to visually assess the dose-response relationship between the four IR surrogates and esophageal cancer. Receiver operating characteristic (ROC) curves and areas under the curve (AUC) were used to assess the discriminatory power of the four IR surrogates in the development of esophageal cancer.

Moreover, subgroup analyses were conducted by stratifying participants based on age, sex, ethnicity, alcohol consumption, smoking status, history of using insulin, DM, CVD, hypertension, MET, and Townsend deprivation index.

All statistical analyses were conducted using R software (version 4.3.1; http://www.R-project.org). All analyses were fully exploratory (hypotheses generating), not confirmatory, and an adjustment for multiple testing was not performed. Two-sided p-values < 0.05 were interpreted as statistical noticeable.

## Results

### Baseline characteristics

Among the final cohort of 388,900 participants, the mean age was 57.0 (50.0–63.0) years, with 47.2% being male and 94.5% being Caucasian. Over a mean follow-up period of 13.0 years, a total of 779 cases of esophageal cancer were recorded, comprising 533 cases of EAC and 202 cases of ESCC. Ten years after recruitment, 0.16% (95%CI 0.11–0.26%) had esophageal cancer and 4.17% (95%CI 3.86–4.46%) are deceased.

In participants with esophageal cancer higher baseline age, male proportion, BMI, history of DM, hypertension, or CVD, and proportions of lipid-lowering medication and insulin use, as well as low levels of four IR surrogates were observed compared to non-esophageal cancer participants (Table 1).

**Table 1:** Baseline demographic and clinical characteristics in the study

Characteristic	Total (*n* = 388,900)	No Esophageal cancer (*n* = 388,121)	Esophageal cancer (*n* = 779)	*P*-value
Age, years	57.0 (50.0–63.0)	57.0 (50.0–63.0)	62.0 (58.0–66.0)	< 0.001
Male, N (%)	183,526 (47.2%)	182,960 (47.1%)	566 (72.7%)	< 0.001
White, N (%)	367,509 (94.5%)	366,742 (94.5%)	767 (98.5%)	< 0.001
MET	2639.0 (1064.0-2895.0)	2639.0 (1064.0-2895.0)	2655.7 (989.8-2847.8)	0.819
Townsend deprivation index	-2.1 (-3.6-0.5)	-2.1 (-3.6-0.5)	-1.7 (-3.4-1.3)	< 0.001
BMI (kg/m2)	26.8 (24.2–29.9)	26.8 (24.2–29.9)	28.1 (25.4–31.2)	< 0.001
Fasting time (h)	3.0 (2.0–4.0)	3.0 (2.0–4.0)	3.0 (3.0–4.0)	0.05
Diet score	5.0 (4.0–6.0)	5.0 (4.0–6.0)	5.0 (4.0–6.0)	< 0.001
DM	20,052 (5.2%)	19,961 (5.1%)	91 (11.7%)	< 0.001
Hypertension, N (%)	107,330 (27.6%)	106,992 (27.6%)	338 (43.4%)	< 0.001
CVD, N (%)	29,817 (7.7%)	29,708 (7.7%)	109 (14.0%)	< 0.001
Lipid lowering drug, N (%)	66,914 (17.2%)	66,668 (17.2%)	246 (31.6%)	< 0.001
Insulin, N (%)	4,183 (1.1%)	4,163 (1.1%)	20 (2.6%)	< 0.001
Alcohol status, N (%)				< 0.001
Never	17,073 (4.4%)	17,049 (4.4%)	24 (3.1%)	
Previous	13,707 (3.5%)	13,662 (3.5%)	45 (5.8%)	
Current	358,120 (92.1%)	357,410 (92.1%)	710 (91.1%)	
Smoking status, N (%)				< 0.001
Never	156,295 (40.2%)	156,104 (40.2%)	191 (24.5%)	
Previous	191,801 (49.3%)	191,362 (49.3%)	439 (56.4%)	
Current	40,804 (10.5%)	40,655 (10.5%)	149 (19.1%)	
TyG index	8.7 (8.3–9.1)	8.7 (8.3–9.1)	8.9 (8.5–9.2)	< 0.001
TyG-BMI	233.5 (204.8-267.6)	233.5 (204.7-267.5)	250.3 (219.5-282.7)	< 0.001
TG/HDL-C	2.4 (1.5-4.0)	2.4 (1.5-4.0)	3.0 (1.9–4.8)	< 0.001
METS-IR	39.0 (33.5–45.5)	39.0 (33.5–45.5)	42.4 (36.3–49.3)	< 0.001

MET: metabolic equivalent task; BMI: body mass index; DM: diabetes mellitus CVD: cardiovascular disease; TyG: triglyceride-glucose; TyG-BMI: TyG index combined with body mass index; TG/HDL-C: triglyceride-to-high-density lipoprotein cholesterol ratio; METS-IR: metabolic score for insulin resistance

### Association of the four IR surrogates with esophageal cancer

**Fig. 2:**
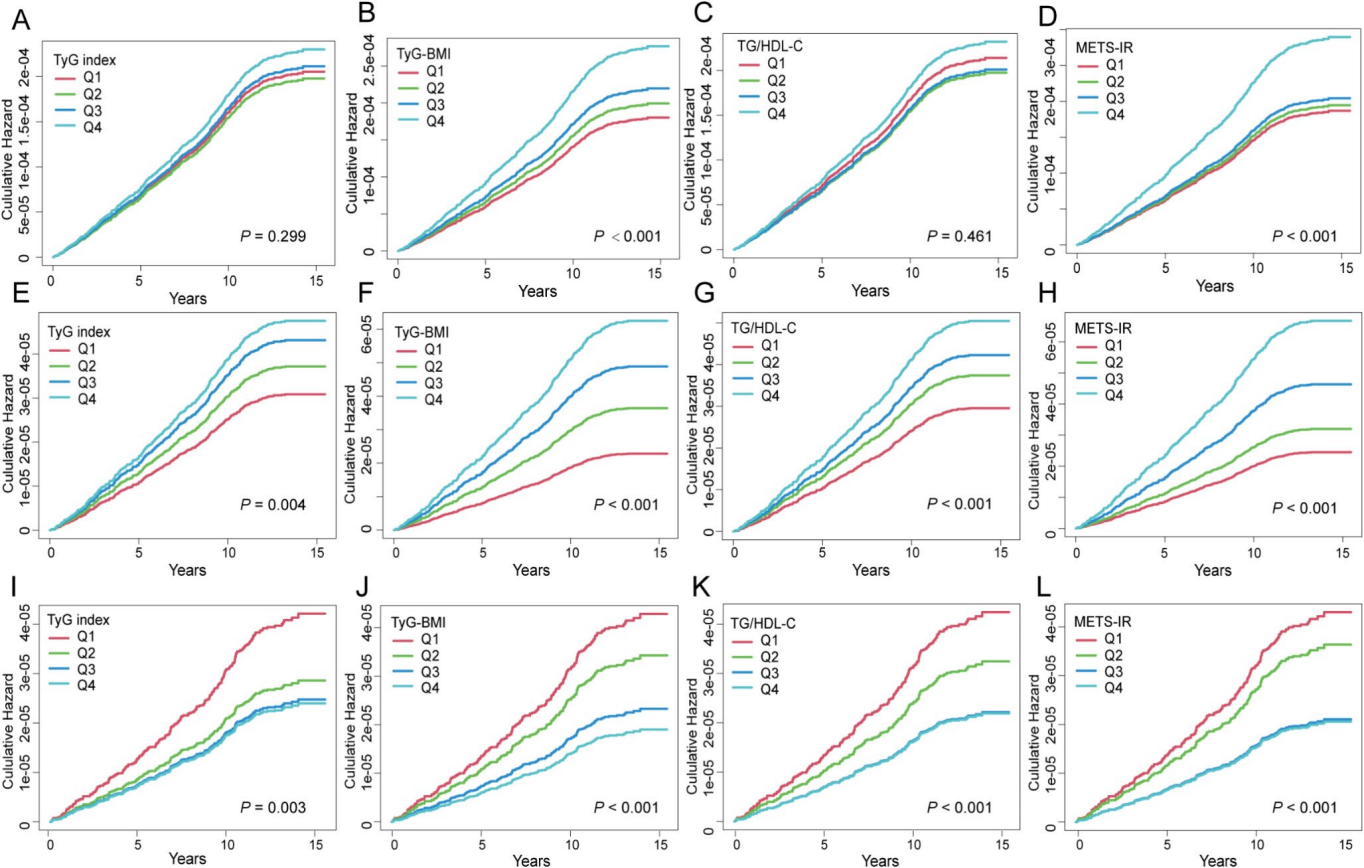
Cumulative incidence for esophageal cancer across the four IR surrogates. **A**–**D**. Esophageal cancer. **E**–**H**. EAC. **I**–**L**, ESCC. Sub-distributional hazard ratios were adjusted for the same variables included in Model 2. EAC: esophageal adenocarcinoma; ESCC: esophageal squamous cell carcinoma. Due to the significant differences between esophageal cancer events and death events, we only plotted the cumulative incidence curve for esophageal cancer events

The four IR surrogates were treated as continuous variables and divided into quartiles, with the first quartile (Q1) serving as a reference. Cumulative incidence functions of esophageal cancer depending on IR surrogates were presented in Fig. 2.

After adjusting for age and sex, the overall risk of esophageal cancer notably increased with increasing TyG index, TyG-BMI, TG/HDL-C, and METS-IR levels (all *P* < 0.05; Table 2). However, in patients with EAC or ESCC, the results for the four IR surrogates varied clearly. Specifically, increasing the TyG index, TyG-BMI, TG/HDL-C, or METS-IR significantly increased the risk of EAC (all *P* < 0.001) and reduced the risk of ESCC (all *P* < 0.05). After comprehensive adjustment for all variables in Model 2, the positive association between TyG-BMI and METS-IR and the overall incidence of esophageal cancer persisted (all *P* < 0.001). However, associations were observed between the risk of esophageal cancer and TyG or TG/HDL-C (both *P* > 0.05). In patients with either EAC or ESCC, increasing TyG index, TyG-BMI, TG/HDL-C, and METS-IR promoted the occurrence of EAC while reducing the risk of ESCC. These HR values (1.16 for TyG index, 1.37 for TyG-BMI, 1.08 for TG/HDL-C, and 1.36 for METS-IR) indicated the increased risk of EAC associated with each SD increase in the respective factors. Conversely, each SD increase in these four IR surrogates (TyG index, TyG-BMI, TG/HDL-C, or METS-IR) was associated with a reduction of ESCC risk with HRs 0.80, 0.67, 0.77 and 0.65, respectively (Table 2). These findings indicate relevant differences in the effects of these four IR surrogates among the different subtypes of esophageal cancer.

**Table 2:** Fine-Gray proportional hazard regression models for the association between four IR surrogates and the risk of esophageal cancer

Type	Model 1			Model 2		
	Esophageal cancer	EAC	ESCC	Esophageal cancer	EAC	ESCC
	HR (95%CI)	HR (95%CI)	HR (95%CI)	HR (95%CI)	HR (95%CI)	HR (95%CI)
**TyG**						
Q1	Reference	Reference	Reference	Reference	Reference	Reference
Q2	1.00 (0.80–1.25)	1.25 (0.91–1.70)	0.69 (0.47-1.00)	0.97 (0.77–1.22)	1.21 (0.88–1.65)	0.67 (0.46–0.97) ^a^
Q3	1.11 (0.89–1.38)	1.51 (1.12–2.02) ^b^	0.61 (0.41–0.90) ^a^	1.04 (0.84–1.29)	1.40 (1.05–1.89) ^a^	0.57 (0.39–0.85) ^b^
Q4	1.31 (1.06–1.61) ^a^	1.80 (1.35–2.38) ^c^	0.60 (0.41–0.89) ^a^	1.13 (0.91–1.40)	1.54 (1.15–2.06) ^b^	0.55 (0.36–0.82) ^b^
P for trend	0.004	< 0.001	0.012	0.160	0.001	0.004
Per SD increase	1.14 (1.07–1.23) ^c^	1.24 (1.14–1.34) ^c^	0.83 (0.71–0.98) ^a^	1.07 (1.00-1.15)	1.16 (1.07–1.26) ^c^	0.80 (0.67–0.95) ^b^
**TyG-BMI**						
Q1	Reference	Reference	Reference	Reference	Reference	Reference
Q2	1.12 (0.88–1.42)	1.64 (1.14–2.35) ^b^	0.82 (0.57–1.18)	1.12 (0.88–1.42)	1.64 (1.14–2.36) ^b^	0.83 (0.57–1.19)
Q3	1.28 (1.02–1.61) ^a^	2.29 (1.63–3.23) ^c^	0.59 (0.40–0.88) ^b^	1.24 (0.99–1.56)	2.22 (1.57–3.13) ^c^	0.57 (0.38–0.85) ^b^
Q4	1.79 (1.44–2.22) ^c^	3.22 (2.31–4.50) ^c^	0.55 (0.37–0.82) ^b^	1.56 (1.24–1.96) ^c^	2.85 (2.02–4.02) ^c^	0.47 (0.31–0.73) ^c^
P for trend	<0.001	< 0.001	0.001	< 0.001	< 0.001	< 0.001
Per SD increase	1.25 (1.17–1.34) ^c^	1.44 (1.34–1.55) ^c^	0.72 (0.59–0.87) ^c^	1.17 (1.08–1.26) ^c^	1.37 (1.26–1.48) ^c^	0.67 (0.55–0.81) ^c^
**TG/HDL-C**						
Q1	Reference	Reference	Reference	Reference	Reference	Reference
Q2	0.96 (0.76–1.21)	1.30 (0.93–1.80)	0.78 (0.54–1.12)	0.93 (0.74–1.17)	1.26 (0.91–1.76)	0.74 (0.52–1.07)
Q3	1.02 (0.82–1.27)	1.52 (1.11–2.08) ^b^	0.55 (0.37–0.83) ^b^	0.95 (0.76–1.18)	1.42 (1.04–1.95) ^c^	0.50 (0.33–0.75) ^c^
Q4	1.26 (1.02–1.56) ^a^	1.91 (1.41–2.59) ^c^	0.56 (0.37–0.86) ^b^	1.10 (0.88–1.36)	1.68 (1.23–2.29) ^c^	0.48 (0.31–0.73) ^c^
P for trend	< 0.001	< 0.001	0.002	0.260	< 0.001	< 0.001
Per SD increase	1.09 (1.03–1.14) ^b^	1.13 (1.07–1.19) ^c^	0.83 (0.66–1.04)	1.03 (0.98–1.09)	1.08 (1.02–1.14) ^a^	0.77 (0.61–0.98) ^a^
**METS-IR**						
Q1	Reference	Reference	Reference	Reference	Reference	Reference
Q2	1.05 (0.82–1.33)	1.32 (0.91–1.91)	0.87 (0.61–1.24)	1.06 (0.83–1.34)	1.34 (0.92–1.93)	0.87 (0.60–1.24)
Q3	1.15 (0.91–1.44)	1.97 (1.40–2.79) ^c^	0.54 (0.36–0.81) ^b^	1.11 (0.88–1.40)	1.94 (1.37–2.74) ^c^	0.51 (0.34–0.76) ^b^
Q4	1.80 (1.45–2.23) ^c^	3.07 (2.21–4.27) ^c^	0.60 (0.41–0.89) ^a^	1.57 (1.25–1.97) ^c^	2.78 (1.98–3.90) ^c^	0.50 (0.32–0.77) ^b^
P for trend	< 0.001	< 0.001	0.002	< 0.001	< 0.001	< 0.001
Per SD increase	1.25 (1.17–1.34) ^c^	1.43 (1.34–1.54) ^c^	0.71 (0.58–0.86) ^c^	1.16 (1.08–1.25) ^c^	1.36 (1.26–1.48) ^c^	0.65 (0.53–0.79) ^c^

HR: hazard ratio. CI: confidence interval. IR: insulin resistance; EAC: esophageal adenocarcinoma; ESCC: esophageal squamous cell carcinoma; SD: standard deviation

Model 1 was adjusted with age, sex; Model 2 were further adjusted with ethnicity, Townsend deprivation index, Metabolic Equivalent of Task (MET), smoking status, alcohol status, diabetes mellitus (DM), hypertension, insulin, fasting time and diet score. a: *P* < 0.05, b: *P* < 0.01, c: *P* < 0.001

### Sensitivity analysis

In the sensitivity analysis, after excluding participants who experienced esophageal cancer events within 2 years (Table S3), the results were consistent with the main findings. In addition, the results remained stable after excluding missing values for all baseline covariates (Table S4), and the results of multiple imputations were consistent with the main results (Table S5 and S6).

### Explorative analyses

**Fig. 3:**
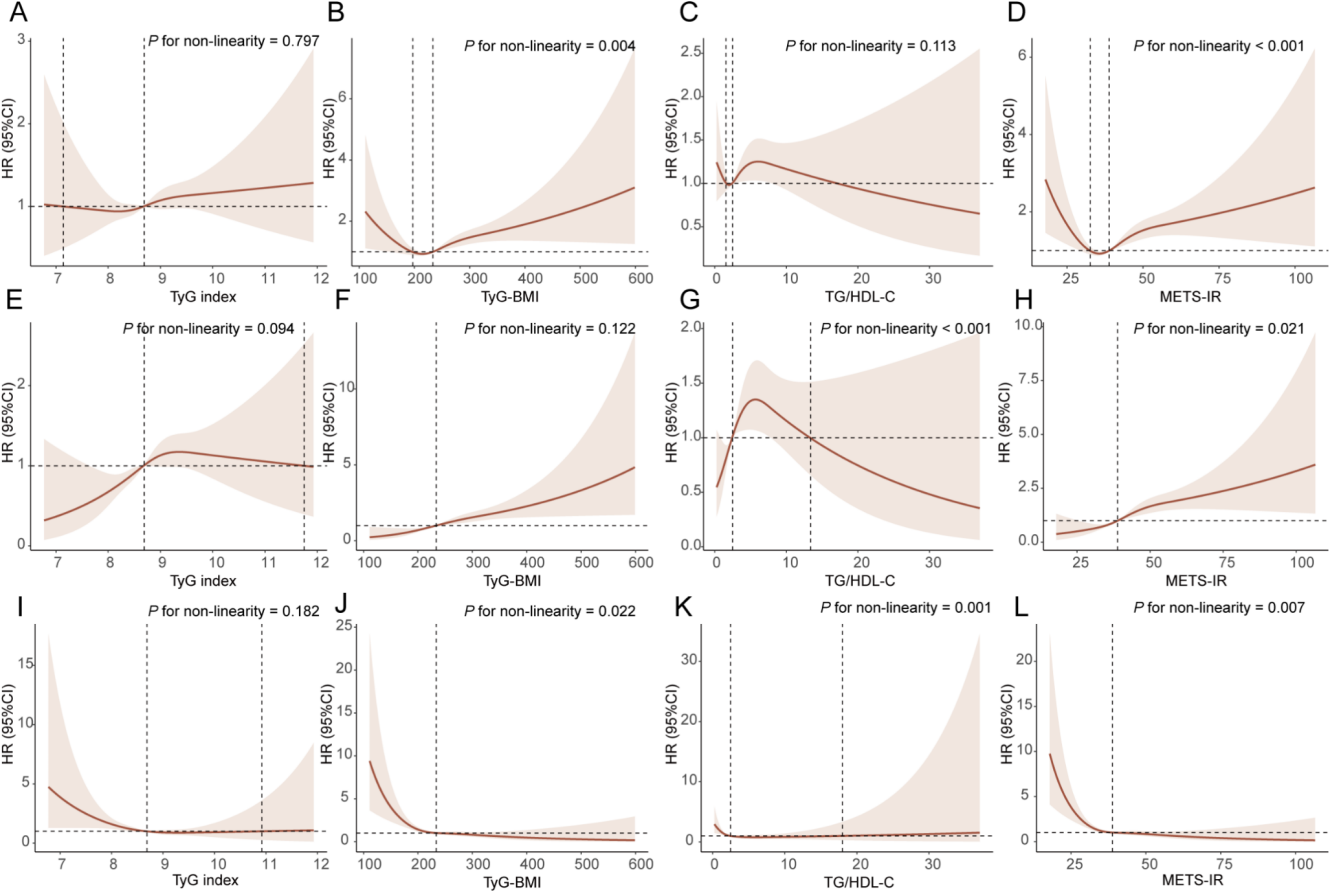
Association of the four IR surrogates with esophageal cancer risk using RCS with 3 knots. **A**–**D**. Esophageal cancer. **E**–**H**. EAC. **I**–**L**, ESCC. Cause-specific hazard ratios were adjusted for the same variables included in Model 2. EAC: esophageal adenocarcinoma; ESCC: esophageal squamous cell carcinoma RCS: restricted cubic spline

After adjusting for maximum covariates, RCS analyses revealed a linear relationship between the TyG index and overall esophageal cancer, EAC, and ESCC (all *P* > 0.05). Additionally, there was a nonlinear relationship between TyG-BMI and EAC (*P* = 0.027) and ESCC (*P* = 0.023). The relationship between TG/HDL-C and either EAC or ESCC was nonlinear (*P* < 0.01). Finally, METS-IR showed a nonlinear relationship with EAC and ESCC (all *P* < 0.05; Fig. 3).

The ROC curve analysis demonstrated that in EAC, METS-IR had the highest AUC (AUC = 0.66; 95%CI 0.64–0.68), followed by TyG-BMI (AUC = 0.64; 95%CI 0.62–0.67), TG/HDL-C ratio (AUC = 0.64; 95%CI: 0.61–0.66), and TyG index (AUC = 0.62; 95%CI 0.60–0.64). Similar results were observed in ESCC (Fig. S2).

### Subgroup analyses

In the subgroup analyses, we observed that the associations of TyG, TyG-BMI, TG/HDL-C, and METS-IR with EAC were more pronounced in female participants, Caucasians, individuals aged < 60 years, smokers, drinkers, and those without baseline chronic diseases, including DM and CVD (Table S7). Similar results were obtained for ESCC (Table S8). Additionally, we found an interaction between TyG-BMI, TG/HDL-C, METS-IR and the risk of EAC when stratified by hypertension (P for interaction < 0.05, Table S7). Similarly, the relationships between TyG-BMI, TG/HDL-C, and METS-IR with ESCC risk were influenced by sex, age, smoking, insulin levels, and cardiovascular disease (P for interaction < 0.05, Table S8).

## Discussion

This prospective cohort study systematically evaluated the associations between four insulin resistance (IR) surrogate markers (TyG index, TyG-BMI, TG/HDL-C, and METS-IR) and the risk of esophageal cancer for the first time. Our findings revealed significant differences in the predictive performance of these IR surrogates for EAC and ESCC.

We observed that elevated levels of TyG-BMI, TG/HDL-C, and METS-IR were significantly associated with an increased risk of EAC and a decreased risk of ESCC. This relationship remained consistent after adjusting for potential confounders, such as age and sex, and was confirmed in further multivariable models. Notably, METS-IR outperformed other IR surrogate markers in predicting both EAC and ESCC, as indicated by its higher area under the ROC curve (AUC), highlighting its potential clinical utility.

Consistent with existing literature, our findings also support the prognostic role of IR surrogate markers in other cancers. For example, the TyG index has been demonstrated to be relevant in breast, ovarian, and prostate cancers (Shi et al. 2022; Jochems et al. 2023). Additionally, the TG/HDL-C ratio has shown similar trends in colorectal and gastric cancer (Kim et al. 2022; Liu et al. 2022). In the case of non-small cell lung cancer (NSCLC), high TyG-BMI levels were associated with poorer survival outcomes, aligning with our findings in esophageal cancer (Wang et al. 2022b).

However, our study also revealed significant differences in the association of different esophageal cancer subtypes to these IR surrogate markers. For instance, while the TyG index and TG/HDL-C were not significantly associated with overall esophageal cancer risk, their varying performance in EAC and ESCC suggests potential biological mechanical differences. Additionally, exploratory analysis indicated a nonlinear relationship between TG/HDL-C and METS-IR with both EAC and ESCC, underscoring the complexity of assessing these markers.

The robustness of our primary findings were further reinforced by sensitivity analyses, which remained consistent even after excluding early esophageal cancer cases and handling missing data. Moreover, subgroup analyses indicated that these associations were more pronounced in specific populations, such as females, smokers, and individuals without chronic diseases, suggesting the potential utility of these markers in different demographic groups.

However, these findings also highlight the need for further research to explore the underlying mechanisms and clinical applications of these markers in esophageal cancer subtypes.

Several potential mechanisms may help explain the inverse carcinogenic effects of the IR index on the development of EAC and ESCC. IR is a metabolic disorder that is often accompanied by obesity, high blood sugar levels, elevated insulin levels, and disturbances in lipid metabolism (Gluvic et al. 2017). Tian et al. showed that being overweight or obese significantly increase the risk of EAC (RR = 1.56; 95%CI 1.42–1.71; *P* < 0.001) (Tian et al. 2020). IR and obesity are closely associated with the occurrence of gastroesophageal reflux disease, long-term acid reflux can damage the esophageal lining, leading to Barrett’s esophagus (a precursor to EAC), thereby increasing the risk of EAC (Budiyani et al. 2018; Schlottmann et al. 2018).

Additionally, IR may mediate cancer progression via the insulin/insulin-like growth factor axis. Human epidermal growth factor receptor 2 (HER2) is a receptor tyrosine kinase closely associated with cell growth and differentiation. Studies have shown that IR activates the HER2 signaling pathway in breast cancer (Shi et al. 2023). The activation of HER2 signaling plays a critical role in the carcinogenesis and progression of EAC (Hu et al. 2011; Creemers et al. 2018; Subasinghe et al. 2018; Plum et al. 2019). Currently, anti-HER2 strategies are important approaches in the treatment of EAC (Janser et al. 2018; Grieb and Agarwal 2021; Liu et al. 2023a). IR is closely associated with MetS and chronic inflammation (Matulewicz and Karczewska-Kupczewska 2016). Prolonged inflammation leads to increased expression of nuclear factor κB (NF-κB), which plays a key role in inflammatory reactions across various tissues, promoting the progression of Barrett’s esophagus and EAC (O’Riordan et al. 2005; Storz et al. 2021). Additionally, hyperinsulinemia is associated with chronic inflammation and cell proliferation, creating an environment conducive to EAC development (Arcidiacono et al. 2018).

Few studies have investigated the relationship between MetS and ESCC. Rothwell et al. indicated a negative correlation between MetS and the risk of ESCC, which is consistent with our findings (Rothwell et al. 2022). However, another study suggests no association between MetS and the risk of ESCC (Lindkvist et al. 2014). Obesity and IR, which are components of MetS, may exert a protective effect in certain cases. Lindkvist et al. found that for each increment of 5 units in BMI, there was a 38% reduction in the risk of developing ESCC (95%CI 0.50–0.79) (Lindkvist et al. 2014). Smith et al. further demonstrated that an increase in BMI significantly improved the prognosis of patients with ESCC (Smith et al. 2008). Similarly, Liu et al. discovered that weight loss is an independent factor associated with poor prognosis in ESCC, whereas diabetes is considered a protective factor for ESCC (Liu et al. 2018). IR is a key feature of obesity and diabetes. Previously, guidelines have reported an inverse association between ESCC and body fatness across various populations (World Cancer Research Fund/American Institute for Cancer Research 2018), which are consistent in our subgroup analyses. The negative correlation between IR and ESCC may be related to body fat percentage and adiposities, insulin resistance, metabolic syndrome, and other underlying biological mechanisms. In our study, an increase in IR indices reduced the risk of developing ESCC – confirming the well-known fact that EAC and ESCC are completely different tumor entities of the esophagus with a completely different pathogenesis.

The strengths of this study include the use of a large sample size in a cohort study with extensive biological and medical data, prospective data collocation, comprehensive long-term follow-up, and adjustment for potential cancer-associated factors. Additionally, this is the first study to investigate this association in patients with esophageal cancer.

However, this study has some limitations. First, primary research questions were defined after data collection and the study design has thus a retrospective character. Especially, variable selection was done data driven without a priori determination.

Second, the four IR indices used in this study were obtained at baseline, making it difficult to infer the dynamic impact of IR index fluctuations on the occurrence of esophageal cancer. Second, this study was based on UKB data, with the majority of participants being Caucasian and lacking validation from external datasets, which limits the generalizability of our findings to other ethnicities and regions. Finally, despite meticulous adjustment for various confounding factors, residual confounding effects could not be excluded.

In conclusion, the TyG index, TyG-BMI, TG/HDL-C, and METS-IR are potential predictive factors of esophageal cancer prevalence. Importantly, an increase in these four IR surrogates may increase the risk of EAC while reducing the risk of ESCC. Therefore, controlling IR surrogates based on the characteristics of esophageal cancer subtypes may be crucial for preventing esophageal cancer and screening high-risk populations.

## Electronic supplementary material

Below is the link to the electronic supplementary material.

Supplementary Material 1

## Data Availability

The datasets generated in this study are available from the corresponding author upon request.
