# Supplementary material for: Association between four insulin resistance surrogates and the risk of esophageal cancer: a prospective cohort study using the UK Biobank
Source: J Cancer Res Clin Oncol. 2024 Aug 24;150(8):399. doi: 10.1007/s00432-024-05919-8 (PMC11344731; doi:10.1007/s00432-024-05919-8)
Supplement: Supplementary file 1 — Supplementary Material 1 [file 432_2024_5919_MOESM1_ESM.docx]

**Association between four insulin resistance surrogates and the risk of esophageal cancer: A retrospective cohort study using the UK Biobank**

**Chuang Yang^1^, Patrick S. Plum^1^, Wenke Cheng^2^, Ines Gockel^1^, eanette Köppe^3^ René Thieme^1*^**

^1^ Department of Visceral, Transplant, Thoracic and Vascular Surgery, University Hospital Leipzig, Liebigstr. 20, D-04103 Leipzig, Germany;

^2^ Medical Faculty, University of Leipzig, Leipzig, Germany;

^3^ Institute of Biostatistics and Clinical Research, University of Muenster, Muenster, Germany

**^*^Correspondence:** rene.thieme@medizin.uni-leipzig.de; Tel.: +49-341-97-20809; Fax: +49-341-97-17209

[Table S1. Definition of diet score in UK Biobank 3](#_Toc170349306)

[Table S2. ICD-10 code and histological subtypes of esophageal cancer in UK Biobank 4](#_Toc170349307)

[Table S3. Sensitivity analysis between the 4 IR surrogates and the risk of EAC and ESCC with participants exclude esophageal cancer occurred within 2 years 5](#_Toc170349308)

[Table S4. Sensitivity analysis between the 4 IR surrogates and the risk of EAC and ESCC with participants exclude all missing values at baseline. 6](#_Toc170349309)

[Table S5. Sensitivity analysis between the 4 IR surrogates and the risk of EAC after multiple imputations of five data sets. 7](#_Toc170349310)

[Table S6. Sensitivity analysis between the 4 IR surrogates and the risk of ESCC after multiple imputations of five data sets. 9](#_Toc170349311)

[Table S7. Subgroup and interaction analysis between the 4 IR surrogates (per SD) and EAC across various subgroups 11](#_Toc170349312)

[Table S8. Subgroup and interaction analysis between the 4 IR surrogates (per SD) and ESCC across various subgroups 13](#_Toc170349313)

[Figure S1. Selection of Covariates by Directed Acyclic Graph 15](#_Toc170349314)

[Figure S2. The discriminatory power of four IR surrogates for the development of esophageal cancer. 16](#_Toc170349315)

| Table S1. Definition of diet score in UK Biobank | | |
| --- | --- | --- |
| **Diet component** | **Criteria** | **Score** |
| Fruit & vegetables | <5 serving/day | 1: If scoring condition met  0: If scoring condition not met (Range: 0-9) |
| Total fish intake | < once a week of each one |  |
| Processed meat | > Once a week |  |
| Red meat | ≤Once a week |  |
| Milk type used | Full cream/ another type of milk/ never rarely have milk |  |
| Spread type | Another selection |  |
| Cereal intake | ≤5 bowls |  |
| Salt added to food | Sometimes/usually/always |  |
| Water intake | < 6 glasses |  |

| Table S2. ICD-10 code and histological subtypes of esophageal cancer in UK Biobank | | | |
| --- | --- | --- | --- |
| **Cancer Type** | **ICD-10 code** | **Histological subtype** | **UK Biobank data field 40011 values** |
| Esophageal cancer | C15.0-15.9 | Adenocarcinoma (EAC) | 8140, 8144, 8210, 8260, 8323, 8480, 8211, 8574, 8481, 8145, 8490 |
|  |  | Squamous cell carcinoma (ESCC) | 8070, 8071 |
|  |  | Other types | 8000, 8010, 8020, 8246, 8560, 8012, 8041, 8720, 8990, 8045 |

Table S3. Sensitivity analysis between the 4 IR surrogates and the risk of EAC and ESCC with participants exclude esophageal cancer occurred within 2 years

| **Type** | **EAC** | **ESCC** |
| --- | --- | --- |
|  | HR (95%CI) | HR (95%CI) |
| **TyG** |  |  |
| Q1 | Reference | Reference |
| Q2 | 1.25 (0.9-1.73) | 0.67 (0.44-1) ^a^ |
| Q3 | 1.48 (1.08-2.02) ^a^ | 0.56 (0.37-0.85) ^b^ |
| Q4 | 1.71 (1.26-2.31) ^c^ | 0.56 (0.37-0.86) ^b^ |
| P for trend | <0.001 | 0.006 |
| Per SD increase | 1.21 (1.10-1.32) ^c^ | 0.81 (0.69-0.96) ^a^ |
| **TyG-BMI** |  |  |
| Q1 | Reference | Reference |
| Q2 | 1.69 (1.16-2.47) ^b^ | 0.95 (0.64-1.4) |
| Q3 | 2.16 (1.50-3.10) ^c^ | 0.60 (0.39-0.93) ^a^ |
| Q4 | 3.09 (2.16-4.41) ^c^ | 0.52 (0.33-0.83) ^b^ |
| P for trend | <0.001 | <0.001 |
| Per SD increase | 1.41 (1.29-1.54) ^c^ | 0.67 (0.55-0.80) ^c^ |
| **TG/HDL-C** |  |  |
| Q1 | Reference | Reference |
| Q2 | 1.31 (0.93-1.85) | 0.73 (0.49-1.08) |
| Q3 | 1.52 (1.10-2.11) ^a^ | 0.52 (0.34-0.8) ^b^ |
| Q4 | 1.77 (1.28-2.43) ^c^ | 0.49 (0.32-0.77) ^b^ |
| P for trend | <0.001 | <0.001 |
| Per SD increase | 1.10 (1.03-1.18) ^b^ | 0.81 (0.66-0.98) ^a^ |
| **METS-IR** |  |  |
| Q1 | Reference | Reference |
| Q2 | 1.47 (1.00-2.15) | 0.96 (0.65-1.41) |
| Q3 | 1.89 (1.31-2.72) ^c^ | 0.53 (0.34-0.83) ^b^ |
| Q4 | 2.93 (2.06-4.18) ^c^ | 0.53 (0.33-0.83) ^b^ |
| P for trend | <0.001 | <0.001 |
| Per SD increase | 1.4 (1.28-1.53) ^c^ | 0.64 (0.53-0.77) ^c^ |

IR: insulin resistance; EAC: esophageal adenocarcinoma; ESCC: esophageal squamous cell carcinoma; SD: standard deviation.

Models were fully adjusted with age, sex, ethnicity, Townsend deprivation index, MET, smoking status, alcohol status, history of diabetes mellitus (DM), hypertension, insulin, fasting time and diet score. a: *P* <0.05, b: *P* <0.01, c: *P* <0.001.

Table S4. Sensitivity analysis between the 4 IR surrogates and the risk of EAC and ESCC with participants exclude all missing values at baseline.

| **Type** | **EAC** | **ESCC** |
| --- | --- | --- |
|  | HR (95%CI) | HR (95%CI) |
| **TyG** |  |  |
| Q1 | Reference | Reference |
| Q2 | 1.35 (0.94-1.95) | 0.49 (0.31-0.77) ^b^ |
| Q3 | 1.54 (1.09-2.18) ^a^ | 0.48 (0.3-0.76) ^b^ |
| Q4 | 1.69 (1.20-2.37) ^b^ | 0.43 (0.26-0.69) ^c^ |
| P for trend | 0.002 | <0.001 |
| Per SD increase | 1.18 (1.06-1.30) ^b^ | 0.7 (0.58-0.85) ^c^ |
| **TyG-BMI** |  |  |
| Q1 | Reference | Reference |
| Q2 | 1.40 (0.93-2.11) | 0.75 (0.48-1.17) |
| Q3 | 1.97 (1.34-2.90) ^c^ | 0.53 (0.32-0.86) ^b^ |
| Q4 | 2.74 (1.87-4.00) ^c^ | 0.43 (0.25-0.72) ^b^ |
| P for trend | <0.001 | <0.001 |
| Per SD increase | 1.42 (1.29-1.56) ^c^ | 0.64 (0.52-0.79) ^c^ |
| **TG/HDL-C** |  |  |
| Q1 | Reference | Reference |
| Q2 | 1.70 (1.15-2.52) ^b^ | 0.83 (0.54-1.27) |
| Q3 | 1.64 (1.12-2.41) ^a^ | 0.40 (0.24-0.67) ^c^ |
| Q4 | 2.12 (1.46-3.07) ^c^ | 0.45 (0.27-0.75) ^b^ |
| P for trend | <0.001 | <0.001 |
| Per SD increase | 1.08 (1.00-1.17) | 0.67 (0.51-0.86) ^b^ |
| **METS-IR** |  |  |
| Q1 | Reference | Reference |
| Q2 | 1.40 (0.91-2.15) | 0.76 (0.49-1.18) |
| Q3 | 1.89 (1.26-2.84) ^b^ | 0.52 (0.31-0.84) ^b^ |
| Q4 | 3.10 (2.08-4.60) ^c^ | 0.43 (0.25-0.73) ^b^ |
| P for trend | <0.001 | <0.001 |
| Per SD increase | 1.41 (1.28-1.55) ^c^ | 0.63 (0.51-0.78) ^c^ |

IR: insulin resistance; EAC: esophageal adenocarcinoma; ESCC: esophageal squamous cell carcinoma; SD: standard deviation.

Models were fully adjusted with age, sex, ethnicity, Townsend deprivation index, MET, smoking status, alcohol status, history of diabetes mellitus (DM), hypertension, insulin, fasting time and diet score. a: *P* <0.05, b: *P* <0.01, c: *P* <0.001.

| Table S5. Sensitivity analysis between the 4 IR surrogates and the risk of EAC after multiple imputations of five data sets. | | | | | | |
| --- | --- | --- | --- | --- | --- | --- |
| **Type** | **EAC** | | | | | |
|  | HR (95%CI) | HR (95%CI) | HR (95%CI) | HR (95%CI) | HR (95%CI) | HR (95%CI) |
|  | Imputation 1 | Imputation 2 | Imputation 3 | Imputation 4 | Imputation 5 | Pooled results |
| **TyG** |  |  |  |  |  |  |
| Q1 | Reference | Reference | Reference | Reference | Reference | Reference |
| Q2 | 1.16 (0.86-1.57) | 1.16 (0.86-1.57) | 1.16 (0.86-1.57) | 1.16 (0.86-1.57) | 1.16 (0.86-1.57) | 1.16 (0.86-1.57) |
| Q3 | 1.39 (1.04-1.84) ^b^ | 1.38 (1.04-1.84) ^a^ | 1.38 (1.04-1.84) ^a^ | 1.38 (1.04-1.84) ^a^ | 1.38 (1.04-1.84) ^a^ | 1.38 (1.04-1.84) ^a^ |
| Q4 | 1.51 (1.14-2) ^c^ | 1.51 (1.14-2) ^b^ | 1.51 (1.14-2) ^b^ | 1.51 (1.14-2) ^b^ | 1.51 (1.14-2) ^b^ | 1.51 (1.14-2) ^b^ |
| P for trend | 0.001 | 0.001 | 0.001 | 0.001 | 0.001 | 0.001 |
| Per SD increase | 1.17 (1.08-1.28) ^c^ | 1.17 (1.08-1.28) ^c^ | 1.17 (1.08-1.28) ^c^ | 1.17 (1.08-1.28) ^c^ | 1.17 (1.08-1.28) ^c^ | 1.17 (1.08-1.28) ^c^ |
| **TyG-BMI** |  |  |  |  |  |  |
| Q1 | Reference | Reference | Reference | Reference | Reference | Reference |
| Q2 | 1.58 (1.11-2.25) ^a^ | 1.58 (1.11-2.25) ^a^ | 1.58 (1.11-2.25) ^a^ | 1.58 (1.11-2.25) ^a^ | 1.58 (1.11-2.25) ^a^ | 1.58 (1.11-2.25) ^a^ |
| Q3 | 2.07 (1.48-2.89) ^c^ | 2.06 (1.48-2.88) ^c^ | 2.07 (1.48-2.89) ^c^ | 2.07 (1.48-2.89) ^c^ | 2.07 (1.48-2.89) ^c^ | 2.07 (1.48-2.89) ^c^ |
| Q4 | 2.82 (2.03-3.93) ^c^ | 2.81 (2.02-3.91) ^c^ | 2.82 (2.03-3.93) ^c^ | 2.82 (2.03-3.93) ^c^ | 2.83 (2.03-3.93) ^c^ | 2.82 (2.03-3.92) ^c^ |
| P for trend | <0.001 | <0.001 | <0.001 | <0.001 | <0.001 | <0.001 |
| Per SD increase | 1.37 (1.26-1.49) ^c^ | 1.37 (1.26-1.49) ^c^ | 1.37 (1.26-1.49) ^c^ | 1.37 (1.26-1.49) ^c^ | 1.37 (1.26-1.49) ^b^ | 1.37 (1.26-1.49) ^c^ |
| **TG/HDL-C** |  |  |  |  |  |  |
| Q1 | Reference | Reference | Reference | Reference | Reference | Reference |
| Q2 | 1.17 (0.86-1.61) | 1.17 (0.85-1.61) | 1.17 (0.86-1.61) | 1.17 (0.85-1.61) | 1.17 (0.86-1.61) | 1.17 (0.85-1.61) |
| Q3 | 1.38 (1.02-1.86) ^a^ | 1.37 (1.02-1.85) ^a^ | 1.38 (1.02-1.86) ^a^ | 1.37 (1.02-1.85) ^a^ | 1.38 (1.02-1.86) ^a^ | 1.38 (1.02-1.86) ^a^ |
| Q4 | 1.61 (1.2-2.16) ^b^ | 1.61 (1.2-2.15) ^b^ | 1.61 (1.2-2.16) ^b^ | 1.61 (1.2-2.15) ^b^ | 1.61 (1.2-2.16) ^b^ | 1.61 (1.2-2.16) ^b^ |
| P for trend | <0.001 | <0.001 | <0.001 | <0.001 | <0.001 | <0.001 |
| Per SD increase | 1.09 (1.02-1.17) ^a^ | 1.09 (1.02-1.17) ^a^ | 1.09 (1.02-1.17) ^b^ | 1.09 (1.02-1.17) ^b^ | 1.09 (1.02-1.17) ^b^ | 1.09 (1.02-1.17) ^a^ |
| **METS-IR** |  |  |  |  |  |  |
| Q1 | Reference | Reference | Reference | Reference | Reference | Reference |
| Q2 | 1.36 (0.95-1.96) | 1.36 (0.95-1.95) | 1.36 (0.95-1.95) | 1.36 (0.95-1.95) | 1.36 (0.95-1.95) | 1.36 (0.95-1.95) |
| Q3 | 1.87 (1.33-2.62) ^c^ | 1.86 (1.33-2.61) ^c^ | 1.87 (1.33-2.62) ^c^ | 1.87 (1.33-2.62) ^c^ | 1.87 (1.33-2.62) ^c^ | 1.87 (1.33-2.62) ^c^ |
| Q4 | 2.78 (2-3.88) ^c^ | 2.77 (1.99-3.86) ^c^ | 2.78 (2-3.87) ^c^ | 2.78 (2-3.87) ^c^ | 2.79 (2-3.88) ^c^ | 2.78 (2-3.87) ^c^ |
| P for trend | <0.001 | <0.001 | <0.001 | <0.001 | <0.001 | <0.001 |
| Per SD increase | 1.37 (1.26-1.49) ^c^ | 1.37 (1.26-1.49) ^c^ | 1.37 (1.26-1.49) ^c^ | 1.37 (1.26-1.49) ^c^ | 1.37 (1.26-1.49) ^c^ | 1.37 (1.26-1.49) ^c^ |

IR: insulin resistance; EAC: esophageal adenocarcinoma; SD: standard deviation.

Models were fully adjusted with age, sex, ethnicity, Townsend deprivation index, MET, smoking status, alcohol status, history of diabetes mellitus (DM), hypertension, insulin, fasting time and diet score. a: *P* <0.05, b: *P* <0.01, c: *P* <0.001.

| Table S6. Sensitivity analysis between the 4 IR surrogates and the risk of ESCC after multiple imputations of five data sets. | | | | | | |
| --- | --- | --- | --- | --- | --- | --- |
| **Type** | **ESCC** | | | | | |
|  | HR (95%CI) | HR (95%CI) | HR (95%CI) | HR (95%CI) | HR (95%CI) | HR (95%CI) |
|  | Imputation 1 | Imputation 2 | Imputation 3 | Imputation 4 | Imputation 5 | Pooled results |
| **TyG** |  |  |  |  |  |  |
| Q1 | Reference | Reference | Reference | Reference | Reference | Reference |
| Q2 | 0.66 (0.45-0.96) ^a^ | 0.66 (0.45-0.96) ^a^ | 0.66 (0.45-0.96) ^a^ | 0.66 (0.45-0.96) ^a^ | 0.66 (0.45-0.96) ^a^ | 0.66 (0.45-0.96) ^a^ |
| Q3 | 0.56 (0.38-0.83) ^b^ | 0.56 (0.38-0.83) ^b^ | 0.56 (0.38-0.83) ^b^ | 0.57 (0.38-0.83) ^b^ | 0.57 (0.38-0.83) ^b^ | 0.56 (0.38-0.83) ^b^ |
| Q4 | 0.54 (0.36-0.8) ^b^ | 0.54 (0.36-0.8) ^b^ | 0.53 (0.36-0.8) ^b^ | 0.54 (0.36-0.81) ^b^ | 0.54 (0.36-0.81) ^b^ | 0.54 (0.36-0.80) ^b^ |
| P for trend | 0.002 | 0.002 | 0.002 | 0.002 | 0.002 | 0.002 |
| Per SD increase | 0.79 (0.68-0.92) ^b^ | 0.79 (0.68-0.92) ^b^ | 0.79 (0.68-0.92) ^b^ | 0.79 (0.68-0.93) ^b^ | 0.79 (0.68-0.93) ^b^ | 0.79 (0.68-0.92) ^b^ |
| **TyG-BMI** |  |  |  |  |  |  |
| Q1 | Reference | Reference | Reference | Reference | Reference | Reference |
| Q2 | 0.81 (0.56-1.16) | 0.81 (0.57-1.17) | 0.81 (0.56-1.16) | 0.81 (0.57-1.17) | 0.81 (0.57-1.17) | 0.81 (0.56-1.16) |
| Q3 | 0.56 (0.37-0.83) ^b^ | 0.56 (0.37-0.83) ^b^ | 0.56 (0.37-0.83) ^b^ | 0.56 (0.38-0.83) ^b^ | 0.56 (0.38-0.83) ^b^ | 0.56 (0.38-0.83) ^b^ |
| Q4 | 0.46 (0.3-0.7) ^c^ | 0.46 (0.3-0.71) ^c^ | 0.46 (0.3-0.7) ^c^ | 0.46 (0.3-0.71) ^c^ | 0.46 (0.3-0.71) ^c^ | 0.46 (0.3-0.71) ^c^ |
| P for trend | <0.001 | <0.001 | <0.001 | <0.001 | <0.001 | <0.001 |
| Per SD increase | 0.66 (0.56-0.78) ^c^ | 0.66 (0.56-0.78) ^c^ | 0.66 (0.56-0.78) ^c^ | 0.66 (0.56-0.78) ^c^ | 0.66 (0.56-0.78) ^c^ | 0.66 (0.56-0.78) ^c^ |
| **TG/HDL-C** |  |  |  |  |  |  |
| Q1 | Reference | Reference | Reference | Reference | Reference | Reference |
| Q2 | 0.74 (0.51-1.05) | 0.74 (0.51-1.06) | 0.73 (0.51-1.05) | 0.74 (0.51-1.06) | 0.74 (0.51-1.06) | 0.74 (0.51-1.06) |
| Q3 | 0.49 (0.33-0.73) ^c^ | 0.49 (0.33-0.73) ^c^ | 0.49 (0.33-0.73) ^c^ | 0.49 (0.33-0.74) ^c^ | 0.49 (0.33-0.74) ^c^ | 0.49 (0.33-0.73) ^c^ |
| Q4 | 0.47 (0.31-0.71) ^c^ | 0.47 (0.31-0.72) ^c^ | 0.47 (0.31-0.71) ^c^ | 0.47 (0.31-0.72) ^c^ | 0.47 (0.31-0.72) ^c^ | 0.47 (0.31-0.71) ^c^ |
| P for trend | <0.001 | <0.001 | <0.001 | <0.001 | <0.001 | <0.001 |
| Per SD increase | 0.77 (0.63-0.93) ^b^ | 0.77 (0.63-0.93) ^b^ | 0.77 (0.63-0.93) ^b^ | 0.77 (0.64-0.93) ^b^ | 0.77 (0.64-0.93) ^b^ | 0.77 (0.64-0.93) ^b^ |
| **METS-IR** |  |  |  |  |  |  |
| Q1 | Reference | Reference | Reference | Reference | Reference | Reference |
| Q2 | 0.85 (0.6-1.22) | 0.85 (0.6-1.22) | 0.85 (0.59-1.22) | 0.85 (0.6-1.22) | 0.85 (0.6-1.22) | 0.85 (0.6-1.22) |
| Q3 | 0.49 (0.33-0.75) ^c^ | 0.5 (0.33-0.75) ^c^ | 0.49 (0.33-0.75) ^c^ | 0.5 (0.33-0.75) ^c^ | 0.5 (0.33-0.75) ^c^ | 0.5 (0.33-0.75) ^c^ |
| Q4 | 0.49 (0.32-0.74) ^c^ | 0.49 (0.32-0.75) ^c^ | 0.48 (0.31-0.74) ^c^ | 0.49 (0.32-0.75) ^c^ | 0.49 (0.32-0.75) ^c^ | 0.49 (0.32-0.75) ^c^ |
| P for trend | <0.001 | <0.001 | <0.001 | <0.001 | <0.001 | <0.001 |
| Per SD increase | 0.64 (0.54-0.76) ^c^ | 0.64 (0.54-0.76) ^c^ | 0.64 (0.54-0.76) ^c^ | 0.64 (0.54-0.76) ^c^ | 0.64 (0.54-0.76) ^c^ | 0.64 (0.54-0.76) ^c^ |

IR: insulin resistance; ESCC: esophageal squamous cell carcinoma; SD: standard deviation.

Models were fully adjusted with age, sex, ethnicity, Townsend deprivation index, MET, smoking status, alcohol status, history of diabetes mellitus (DM), hypertension, insulin, fasting time and diet score. a: *P* <0.05, b: *P* <0.01, c: *P* <0.001.

| Table S7. Subgroup and interaction analysis between the 4 IR surrogates (per SD) and EAC across various subgroups | | | | | | | | |
| --- | --- | --- | --- | --- | --- | --- | --- | --- |
| **Subgroups** | **TyG** | | **TyG-BMI** | | **TG/HDL-C** | | **METS-IR** | |
|  | HR (95% Cl) | P for interaction | HR (95% Cl) | P for interaction | HR (95% Cl) | P for interaction | HR (95% Cl) | P for interaction |
| Sex |  | 0.479 |  | 0.547 |  | 0.425 |  | 0.414 |
| Men | 1.16 (1.06-1.27) ^b^ |  | 1.33 (1.21-1.46) ^c^ |  | 1.09 (1.02-1.17) ^a^ |  | 1.33 (1.21-1.46) ^c^ |  |
| Women | 1.29 (1.03-1.63) ^a^ |  | 1.48 (1.23-1.77) ^c^ |  | 1.22 (0.97-1.53) |  | 1.51 (1.26-1.83) ^c^ |  |
| Race |  | 0.400 |  | 0.674 |  | 0.357 |  | 0.793 |
| White | 1.18 (1.08-1.29) ^c^ |  | 1.36 (1.25-1.48) ^c^ |  | 1.10 (1.03-1.18) ^b^ |  | 1.36 (1.25-1.48) ^c^ |  |
| Other | 0.86 (0.34-2.19) |  | 1.34 (0.57-3.10) |  | 0.70 (0.18-2.69) |  | 1.25 (0.52-3.02) |  |
| Age |  | 0.920 |  | 0.174 |  | 0.984 |  | 0.168 |
| <60 | 1.15 (1.00-1.33) ^a^ |  | 1.43 (1.26-1.63) ^c^ |  | 1.09 (0.98-1.21) |  | 1.44 (1.27-1.64) ^c^ |  |
| >=60 | 1.18 (1.06-1.32) ^b^ |  | 1.29 (1.15-1.44) ^c^ |  | 1.10 (1.01-1.20) ^a^ |  | 1.30 (1.16-1.45) ^c^ |  |
| Alcohol |  | 0.198 |  | 0.689 |  | 0.415 |  | 0.890 |
| No | 0.69 (0.36-1.32) |  | 1.04 (0.6-1.81) |  | 0.70 (0.36-1.39) |  | 1.09 (0.63-1.86) |  |
| Yes | 1.19 (1.09-1.30) ^c^ |  | 1.36 (1.25-1.49) ^c^ |  | 1.10 (1.03-1.18) ^b^ |  | 1.37 (1.26-1.49) ^c^ |  |
| Smoking |  | 0.489 |  | 0.935 |  | 0.212 |  | 0.921 |
| Yes | 1.21 (1.10-1.33) ^c^ |  | 1.37 (1.25-1.51) ^c^ |  | 1.12 (1.05-1.20) ^b^ |  | 1.38 (1.26-1.52) ^c^ |  |
| No | 1.07 (0.89-1.28) |  | 1.30 (1.09-1.55) ^b^ |  | 0.98 (0.82-1.18) |  | 1.28 (1.07-1.53) ^b^ |  |
| Insulin |  | 0.282 |  | 0.694 |  | 0.761 |  | 0.760 |
| Yes | 1.40 (1.00-1.98) |  | 1.25 (0.86-1.80) |  | 1.06 (0.82-1.37) |  | 1.27 (0.90-1.80) |  |
| No | 1.16 (1.06-1.27) ^c^ |  | 1.36 (1.25-1.49) ^c^ |  | 1.10 (1.03-1.18) ^b^ |  | 1.37 (1.26-1.49) ^c^ |  |
| DM |  | 0.301 |  | 0.343 |  | 0.054 |  | 0.256 |
| Yes | 1.05 (0.87-1.27) |  | 1.16 (0.95-1.41) |  | 0.93 (0.77-1.12) |  | 1.15 (0.94-1.39) |  |
| No | 1.21 (1.10-1.33) ^c^ |  | 1.4 (1.27-1.54) ^c^ |  | 1.14 (1.06-1.23) ^c^ |  | 1.41 (1.29-1.55) ^c^ |  |
| MET |  | 0.407 |  | 0.446 |  | 0.953 |  | 0.593 |
| <mean (2655.709) | 1.19 (1.05-1.34) ^b^ |  | 1.36 (1.21-1.53) ^c^ |  | 1.09 (0.99-1.19) |  | 1.36 (1.21-1.52) ^c^ |  |
| >= mean (2655.709) | 1.16 (1.03-1.32) ^a^ |  | 1.34 (1.18-1.51) ^c^ |  | 1.11 (1.01-1.22) ^a^ |  | 1.35 (1.2-1.53) ^c^ |  |
| Townsend |  | 0.475 |  | 0.567 |  | 0.326 |  | 0.578 |
| < mean (-1.301) | 1.21 (1.08-1.36) ^b^ |  | 1.38 (1.22-1.55) ^c^ |  | 1.13 (1.04-1.24) ^b^ |  | 1.39 (1.23-1.56) ^c^ |  |
| >=mean (-1.301) | 1.14 (1.00-1.29) ^a^ |  | 1.34 (1.19-1.51) ^c^ |  | 1.06 (0.96-1.17) |  | 1.34 (1.19-1.51) ^c^ |  |
| CVD |  | 0.277 |  | 0.518 |  | 0.192 |  | 0.490 |
| Yes | 1.03 (0.82-1.29) |  | 1.26 (1.01-1.58) ^a^ |  | 0.97 (0.80-1.18) |  | 1.26 (1.02-1.57) ^a^ |  |
| No | 1.20 (1.09-1.32) ^c^ |  | 1.37 (1.25-1.51) ^c^ |  | 1.12 (1.04-1.20) ^b^ |  | 1.38 (1.02-1.57) ^c^ |  |
| Hypertension |  | 0.051 |  | 0.025 |  | 0.020 |  | 0.009 |
| Yes | 1.05 (0.93-1.19) |  | 1.22 (1.08-1.38) ^b^ |  | 0.99 (0.89-1.11) |  | 1.20 (1.06-1.35) ^b^ |  |
| No | 1.30 (1.16-1.46) ^c^ |  | 1.52 (1.35-1.70) ^c^ |  | 1.18 (1.10-1.28) ^c^ |  | 1.55 (1.38-1.74) ^c^ |  |

IR: insulin resistance; EAC: esophageal adenocarcinoma; SD: standard deviation.

Models were fully adjusted with age, sex, ethnicity, Townsend deprivation index, MET, smoking status, alcohol status, history of diabetes mellitus (DM), hypertension, insulin, fasting time and diet score. a: *P* <0.05, b: *P* <0.01, c: *P* <0.001.

| Table S8. Subgroup and interaction analysis between the 4 IR surrogates (per SD) and ESCC across various subgroups | | | | | | | | |
| --- | --- | --- | --- | --- | --- | --- | --- | --- |
| **Subgroups** | **TyG** | | **TyG-BMI** | | **TG/HDL-C** | | **METS-IR** | |
|  | HR (95% Cl) | P for interaction | HR (95% Cl) | P for interaction | HR (95% Cl) | P for interaction | HR (95% Cl) | P for interaction |
| Sex |  | 0.977 |  | 0.011 |  | 0.43 |  | 0.012 |
| Men | 0.79 (0.64-0.99) ^a^ |  | 0.83 (0.65-1.07) |  | 0.84 (0.66-1.05) |  | 0.81 (0.63-1.04) |  |
| Women | 0.81 (0.65-1.01) |  | 0.54 (0.43-0.68) ^c^ |  | 0.74 (0.54-1.01) |  | 0.52 (0.41-0.67) ^c^ |  |
| Race |  | 0.236 |  | 0.170 |  | 0.781 |  | 0.266 |
| White | 0.79 (0.68-0.93) ^b^ |  | 0.64 (0.53-0.76) ^c^ |  | 0.80 (0.66-0.97) ^a^ |  | 0.63 (0.52-0.75) ^c^ |  |
| Other | 1.11 (0.54-2.28) |  | 1.15 (0.53-2.49) |  | 0.80 (0.31-2.05) |  | 1.04 (0.46-2.35) |  |
| Age |  | 0.128 |  | 0.099 |  | 0.014 |  | 0.064 |
| <60 | 0.86 (0.67-1.11) |  | 0.72 (0.55-0.94) ^a^ |  | 0.95 (0.75-1.22) |  | 0.72 (0.54-0.94) ^a^ |  |
| >=60 | 0.75 (0.62-0.92) ^b^ |  | 0.61 (0.49-0.75) ^c^ |  | 0.67 (0.51-0.88) ^b^ |  | 0.59 (0.47-0.74) ^c^ |  |
| Alcohol |  | 0.378 |  | 0.303 |  | 0.904 |  | 0.285 |
| No | 1.07 (0.58-1.96) |  | 0.50 (0.25-1.01) |  | 1.00 (0.52-1.92) |  | 0.54 (0.26-1.10) |  |
| Yes | 0.79 (0.67-0.92) ^b^ |  | 0.66 (0.56-0.79) ^c^ |  | 0.79 (0.65-0.96) ^a^ |  | 0.65 (0.54-0.77) ^c^ |  |
| Smoking |  | 0.140 |  | 0.935 |  | 0.013 |  | 0.572 |
| Yes | 0.87 (0.73-1.04) |  | 0.66 (0.54-0.80) ^c^ |  | 0.88 (0.72-1.07) |  | 0.65 (0.53-0.80) ^c^ |  |
| No | 0.65 (0.48-0.88) ^b^ |  | 0.63 (0.46-0.87) ^b^ |  | 0.49 (0.29-0.83) ^b^ |  | 0.60 (0.43-0.84) ^b^ |  |
| Insulin |  | 0.324 |  | 0.050 |  | 0.016 |  | 0.057 |
| Yes | 0.53 (0.24-1.19) |  | 0.17 (0.03-0.88) ^a^ |  | 0.02 (0.00-3.10) |  | 0.17 (0.03-0.96) ^a^ |  |
| No | 0.82 (0.7-0.96) ^a^ |  | 0.67 (0.56-0.80) ^c^ |  | 0.82 (0.68-0.99) ^a^ |  | 0.66 (0.55-0.78) ^c^ |  |
| DM |  | 0.601 |  | 0.519 |  | 0.101 |  | 0.540 |
| Yes | 0.73 (0.46-1.16) |  | 0.56 (0.32-0.98) ^a^ |  | 0.45 (0.19-1.04) |  | 0.56 (0.32-0.98) ^a^ |  |
| No | 0.82 (0.7-0.96) ^a^ |  | 0.66 (0.55-0.79) ^c^ |  | 0.84 (0.69-1.02) |  | 0.65 (0.54-0.78) ^c^ |  |
| MET |  | 0.052 |  | 0.683 |  | 0.078 |  | 0.606 |
| <mean (2655.709) | 0.63 (0.5-0.80) ^c^ |  | 0.62 (0.48-0.80) ^c^ |  | 0.60 (0.43-0.83) ^b^ |  | 0.61 (0.47-0.80) ^c^ |  |
| >=mean (2655.709) | 0.97 (0.79-1.19) |  | 0.66 (0.53-0.83) ^c^ |  | 0.95 (0.76-1.19) |  | 0.64 (0.51-0.82) ^c^ |  |
| Townsend |  | 0.183 |  | 0.904 |  | 0.272 |  | 0.888 |
| <mean (-1.301) | 0.90 (0.72-1.13) |  | 0.66 (0.51-0.85) ^b^ |  | 0.93 (0.73-1.20) |  | 0.67 (0.52-0.87) ^b^ |  |
| >=mean (-1.301) | 0.73 (0.59-0.90) ^b^ |  | 0.65 (0.52-0.82) ^c^ |  | 0.69 (0.52-0.91) ^b^ |  | 0.62 (0.49-0.79) ^c^ |  |
| CVD |  | 0.101 |  | 0.005 |  | 0.529 |  | 0.014 |
| Yes | 0.56 (0.38-0.84) ^b^ |  | 0.32 (0.2-0.53) ^c^ |  | 0.64 (0.38-1.05) |  | 0.33 (0.21-0.55) ^c^ |  |
| No | 0.86 (0.73-1.02) |  | 0.73 (0.61-0.87) ^c^ |  | 0.83 (0.68-1.02) |  | 0.71 (0.59-0.85) ^c^ |  |
| Hypertension |  | 0.296 |  | 0.411 |  | 0.479 |  | 0.558 |
| Yes | 0.74 (0.58-0.95) ^a^ |  | 0.61 (0.47-0.79) ^c^ |  | 0.74 (0.55-1.00) |  | 0.61 (0.47-0.79) ^c^ |  |
| No | 0.84 (0.69-1.03) |  | 0.68 (0.54-0.85) ^c^ |  | 0.83 (0.66-1.06) |  | 0.66 (0.52-0.83) ^c^ |  |

IR: insulin resistance; ESCC: esophageal squamous cell carcinoma; SD: standard deviation.

Models were fully adjusted with age, sex, ethnicity, Townsend deprivation index, MET, smoking status, alcohol status, history of diabetes mellitus (DM), hypertension, insulin, fasting time and diet score. a: *P* <0.05, b: *P* <0.01, c: *P* <0.001.


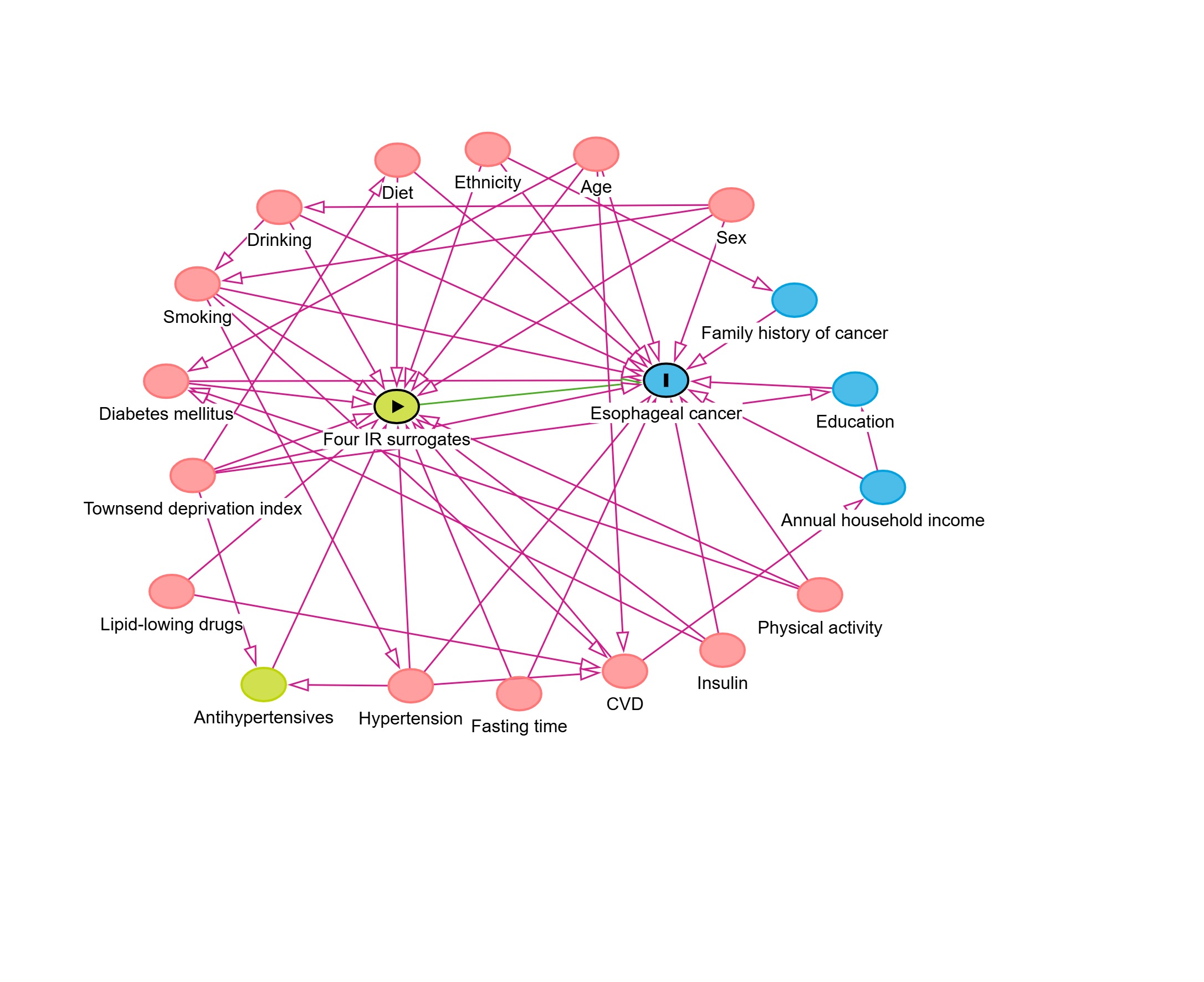


**Figure S1. Selection of Covariates by Directed Acyclic Graph**

Diagram established by online tools (https://www.dagitty.net/). Minimal adjustment set age, sex, ethnicity, Townsend deprivation index, MET, smoking status, alcohol status, history of diabetes mellitus, hypertension, insulin, fasting time and diet score.

.


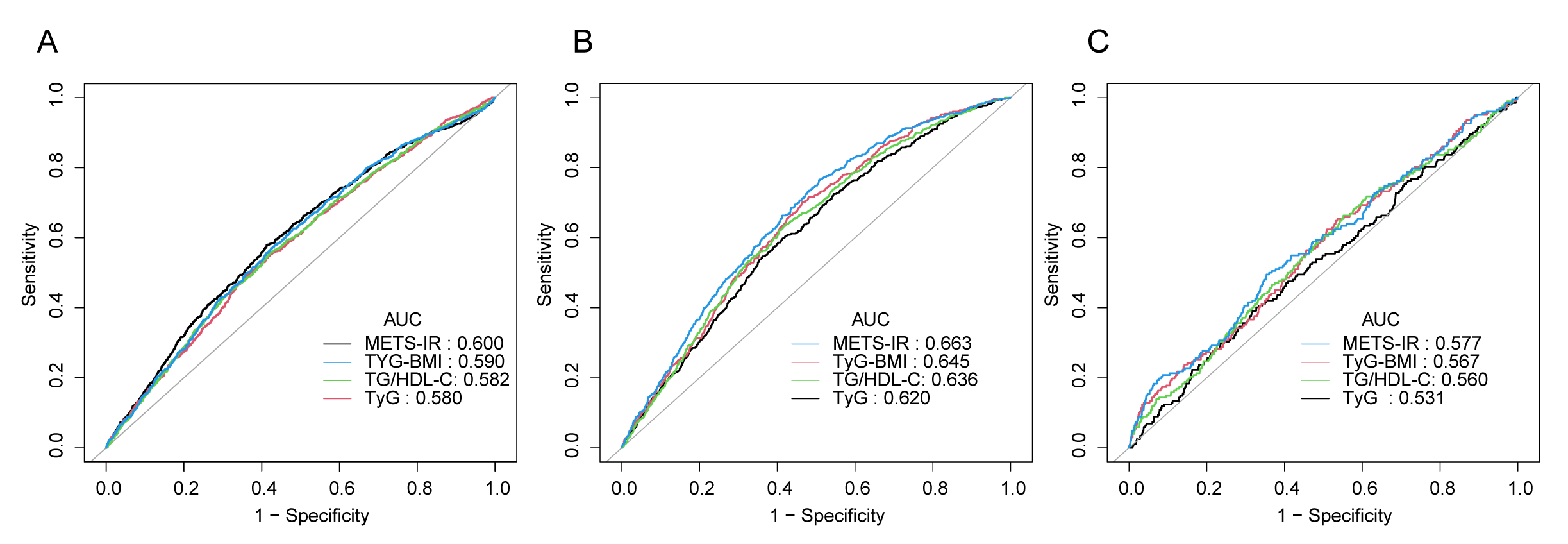


Figure S2. The discriminatory power of four IR surrogates for the development of esophageal cancer. A. Esophageal cancer. B. EAC. C. ESCC.
